# Insight Into Neonatal Sepsis: An Overview

**DOI:** 10.7759/cureus.45530

**Published:** 2023-09-19

**Authors:** Hussein Attia Hussein Mahmoud, Ritika Parekh, Sarvani Dhandibhotla, Tarun Sai, Aayush Pradhan, Shiny Alugula, Martin Cevallos-Cueva, Barbara K Hayes, Saranya Athanti, Zain Abdin, Basant K

**Affiliations:** 1 Diagnostic Radiology, Heliopolis Hospital, Cairo, EGY; 2 Community Health, K. J. Somaiya Medical College and Research Centre, Mumbai, IND; 3 Gastroenterology, Massachusetts General Hospital, Boston, USA; 4 Pediatrics, Sri Balaji Medical College Hospital and Research Institute, Tirupati, IND; 5 Pediatric Emergency, International Friendship Children’s Hospital, Kathmandu, NPL; 6 Pediatric Surgery, New Vision University, Eluru, IND; 7 Pediatrics, Universidad Central del Ecuador, Quito, ECU; 8 Pediatric Intensive Care Unit, Suburbio Hospital, Salvador, BRA; 9 Pediatrics, Employees' State Insurance Corporation Medical College and Hospital, Hyderabad, IND; 10 Critical Care Medicine, IMG (International Medical Graduate) Helping Hands, Albuquerque, USA; 11 Medicine, Tanta University, Tanta, EGY

**Keywords:** overview, pediatrics, infection, neonatal sepsis, neonates

## Abstract

There are approximately 1.3 million cases of neonatal sepsis reported worldwide with deaths occurring more commonly in preterm and low-weight newborns. Neonatal sepsis is the third major cause of neonatal deaths resulting in 203,000 deaths per year. It is divided into two subtypes based on time of occurrence: early-onset neonatal sepsis (ENS), occurring within the first 72 hours of birth usually due to perinatal risk factors, and late-onset neonatal sepsis (LOS) usually occurring after the first week of life and up to 28th day of life. There are many complications associated with neonatal sepsis including septic shock, multiple organ failure, and death. It is vital for clinicians to know the signs and symptoms of neonatal sepsis in order to diagnose it early. Preventive measures, early diagnosis, appropriate antibiotic administration, timely supportive management, and the establishment of efficient management are vital in the prevention of severe complications or death. In this review, we aim to provide the most up-to-date information regarding risk factors, pathophysiology, signs and symptoms, diagnosis, and treatment of neonatal sepsis. We discuss the maternal and neonatal risk factors involved in the pathogenesis of neonatal sepsis and the signs and symptoms of early and late neonatal sepsis. We focus on the different pathogens involved and the markers used in the diagnosis and treatments available for each.

## Introduction and background

Introduction

Neonatal sepsis (NS) is a dysregulated host response to a systemic viral, bacterial, or fungal infection in the first 28 days of life that is potentially fatal and could turn life-threatening in both term and preterm newborns [1]. Neonatal sepsis is categorized into two major groups: early-onset sepsis (EOS) and late-onset sepsis (LOS) depending on the time of infection, mode of transmission, and causative organisms [2]. EOS describes a vertically transmitted infection in the first three days of life (72 hours), and LOS is a horizontally transmitted infection (after 72 hours of life) commonly caused by a microorganism in hospital settings. Although some researchers consider seven days as a cutoff limit for differentiating EOS and LOS, most epidemiological studies recommend 72 hours as a reference [3]. Moreover, a third group, very late-onset neonatal sepsis (VLOS), has also been described by some as a third classification. VLOS mainly occurs in infants hospitalized in the neonatal intensive care unit (NICU); it usually occurs after 30 days of hospitalization till discharge [4].

Aims and clinical relevance

Neonatal sepsis is responsible for approximately 8% of neonates' deaths and is a predominant cause of neonatal mortality and long-term morbidity, especially in low- and middle-income countries [5,6]. Although the epidemiology of NS is constantly changing, approximately 1.3 to 3.9 million new cases are reported annually by the Global Burden of Disease (GBD). An estimated 24% of deaths among this vulnerable group of infants are caused by severe infections [5,6]. A recent systematic review and meta-analysis indicated that the approximate EOS incidence is 2,496 per 100,000 live births, which was 2.6 times more common than LOS, 946 per 100 live births [7]. However, the overall incidence of EOS has been declining throughout the years, from 1990 to 2015, because of universal group B streptococcus screening and intrapartum antibiotic prophylaxis. Incidence has declined from 1.37 to 0.23 per 1,000 live births. Nonetheless, the incidence of LOS has remained nearly unchanged, at 0.31 per 1,000 live births [8,9]. The prevalence of sepsis is significantly higher in both preterm and low-weight newborns, with a reported mortality of 17.6% [5].

## Review

Pathophysiology

Neonatal sepsis is a clinical syndrome that is characterized by signs and symptoms of infection usually associated with bacteremia, which leads to a systemic inflammatory response syndrome that further leads to multiorgan dysfunction [10,11]. Early-onset neonatal sepsis includes gram-positive bacteria like *Streptococcus agalactiae* and *Escherichia coli*, *Staphylococcus aureus*, *Enterococcus*, and *Streptococcus pneumonia* [2]. Late-onset neonatal sepsis includes gram-negative bacteria, coagulase-negative staphylococci (CONS), *Klebsiella pneumoniae*, *Acinetobacter baumannii* [2,12], and viral pathogens which include echovirus, enterovirus, parechovirus, coxsackie virus, adenovirus, parainfluenza virus, rhinovirus, and coronavirus [13]. Fungal causes are uncommon, with the most common fungal cause being Candida [13].

Maternal risk factors include prolonged rupture of membranes, chorioamnionitis, and poor prenatal care. Neonatal risk factors include prematurity of the fetal immune system, congenital dermatologic abnormality, and birth asphyxia which disrupts host defenses and predisposes to infection [10]. Moreover, preterm infants are shown to be exposed to bacteria in utero, while term infants are most probably exposed to bacteria in the birth canal during labor [14]. During the early stage of sepsis, host immunity plays a major role in pathogenesis, and the innate immune system acts as a defense, while adaptive immunity still requires maturity. Innate barriers are skin and mucosal barriers that act by producing acidic pH, mucus, and cilia [15]. However, neonates have less acid production and motility and low-level productive mucous; respiratory epithelia produce mucociliary clearance. In preterm neonates, there are more goblet cells compared to normal and the reduced mucociliary clearance results in an inability to clear bacterial debris; thus, they are prone to sepsis [16]. Gastrointestinal epithelia including Paneth and intestinal lymphoid cells produce interleukin 17 that helps activate adaptive immunity [16].

**Figure 1:**
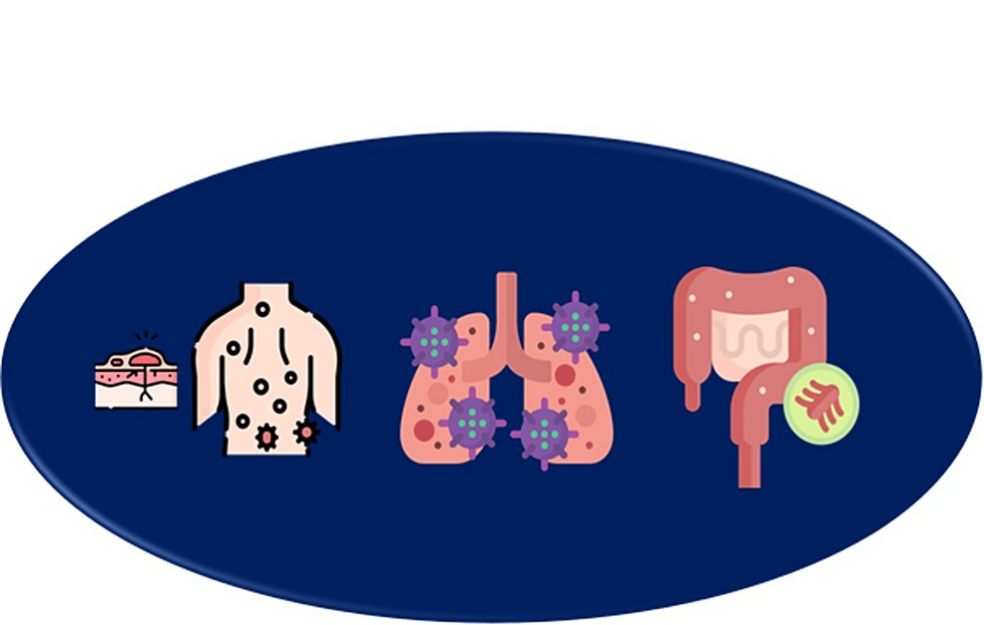
Risk factors and portal of entry

**Figure 2:**
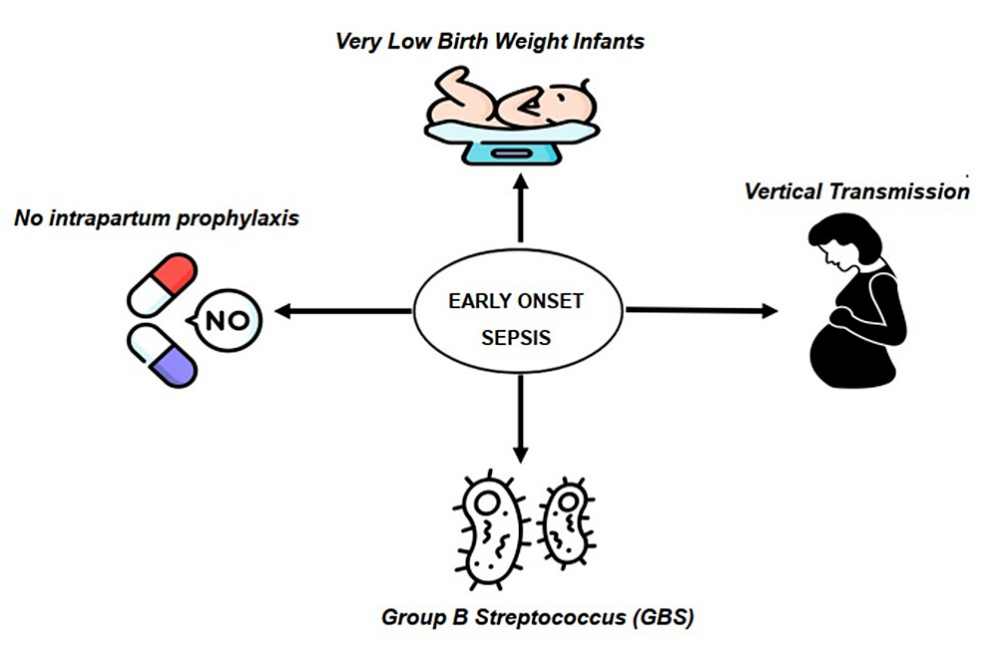
Most common causes of early-onset sepsis

**Figure 3:**
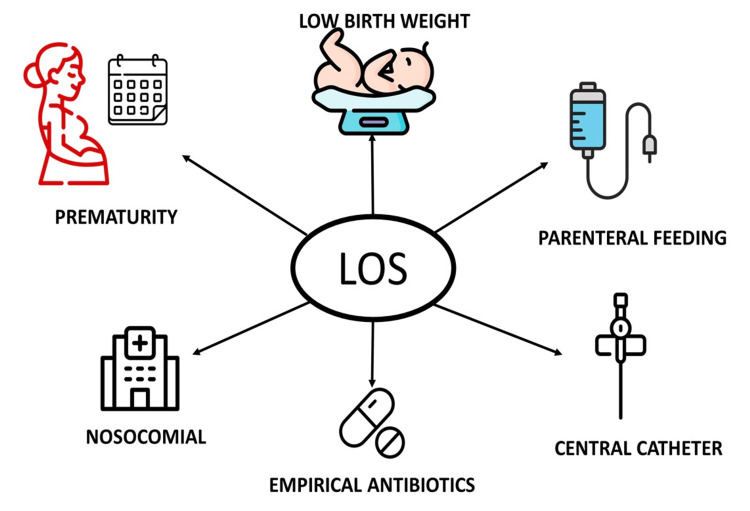
Most common causes of late-onset sepsis LOS: late-onset sepsis.

EOS pathophysiology can be categorized from least to most common as follows: retroperitoneal accession via the fallopian tube, vertical bacterial transmission from the mother before birth (when pathogens from the vagina ascend to the uterus and reach the fetus through hematogenous transmission), and contamination of the fetus's mucous membranes during vaginal birth by microorganisms from the birth canal, maternal genitourinary tract colonizers, and perineal area, potentially affecting the lungs or intestines [17-19]. Risk factors are summarized in Figure 1. The most common causes of EOS and LOS are summarized in Figure 2 and Figure 3, respectively.

Role of invasion

Infection occurs in the fetus via vertical transmission of maternal bacteria from the lower genital tract to the uterus. This contaminates the amniotic fluid leading to hematogenous transmission causing fetal bacteremia and sepsis [20]. Infection may be caused by gram-negative or gram-positive bacteria. The gram-negative bacterial wall is made of endotoxin lipopolysaccharide (LPS), while the gram-positive is made of exotoxin lipoteichoic acid (LTA) [21]. LPS sends signals via Toll-like receptor 4 (TLR-4), and lipopolysaccharide-binding protein (LBP) exhibits an important role as a biomarker. In response to infection, LBP detects microbial-associated molecular patterns of bacteria to deliver endotoxin to CD14 immune effector cells [22]. In addition, lipoteichoic acid is secreted by gram-positive bacterial cell wall signal via Toll-like receptor 2 (TLR-2) [10,21,23].

Vascular endothelium and sentinel cells also play a role (such as monocytes and macrophages); they detect infections by the binding of pathogen-associated molecular patterns (PAMPs) (LPS, lipoteichoic acid (LTA), DNA, RNA) to pathogen recognition receptors (PRRs) (TLRs, Rig-1-like receptors (RLRs), Nod-like receptors (NLRs)) [24-26]. As a result, the transcription of the nuclear factor kappa-light-chain-enhancer of activated B cells (NF-kB) is shifted into the cell nucleus because of signal transduction caused by the binding of PAMPs and damage-associated molecular patterns (DAMPs) to TLRs on granulocytes, antigen-presenting cells ((APCs) like dendritic cells, macrophages), and monocytes. Those cells also act as secondary mediators which causes cytokine storm [26]. This causes an early proinflammatory response to pathogen invasion defined by the activation of cytokines, interleukins, chemokines, growth factor, tumor necrosis factor, and interferon by the activated innate immune system, but neonates have an immature function of macrophages, polymorphonuclear cells, and T cells, so results in incomplete inflammatory actions [27]. In response to these proinflammatory cytokines, LPS, and DAMPs (which are host cell components released during lysis), the vascular endothelial activates and results in the upregulation of polymorphonuclear neutrophils (PMNs) and leukocyte trafficking. Coagulation/thrombolysis/fibrinolysis caused by the activated immune system and pro-inflammatory mediators activates lymphocytes resulting in a coagulation cascade [28] and increased vascular permeability [29,30].

Shedding of the endothelial glycocalyx in the vascular endothelium is affected by proinflammatory cytokines, and upstream mediators like reactive oxygen species, proteoglycans, glycosaminoglycan, and derma sulfate are implicated by activating sheddase enzymes like heparanase1, heparanase 2, hyaluronidase, and metalloproteinase [23] that cleave transmembrane proteins. This plays a primary role in the pathophysiology by causing dysfunction of hemostasis [27]. Microvascular thrombosis leads to an increase in leukocyte adhesion (platelet adhesion), nitric oxide vasodilation leads to abnormal function of vascular tone, and hyperpermeability leads to tissue edema; this microcirculatory dysfunction leads to hypoperfusion and multiorgan dysfunction.

Signs and symptoms

Clinical identification of neonatal sepsis is very challenging as compared to pediatric or adult kind due to vague and nonspecific clinical signs with the inconspicuous and overlapping non-sepsis syndrome in this age group [31,32]. These include severe dehydration, acute respiratory distress syndrome (ARDS), cyanotic congenital heart disease (CCHD), meconium aspiration syndrome, and any congenital cause of bowel obstruction or metabolic disorder that can present as neonatal sepsis. Signs and symptoms of neonatal sepsis are variable; however, early signs commonly include fever or hypothermia, tachypnea (i.e., increased respiration), tachycardia (increased heart rate), poor feeding, inconsolable child, and lethargy all warrant consideration of sepsis [33]. Studies have found abnormal heart rates and a decrease in variability and transient decelerations. They were noted to occur 14 hours prior to the onset of symptoms [34]. Similarly, another study also showed an asymmetric increase in the respiratory rate (RR) interval occurring three to four days before sepsis [35]. The most common signs and symptoms of both early and late neonatal sepsis are listed in Table 1.

**Table 1:** Signs and symptoms of neonatal sepsis

System	Early Sepsis	Late Sepsis
Cardiac	Tachycardia, hypotension	Bradycardia, delayed capillary refill, diminished pulses
Respiratory	Tachypnea, nasal flaring	Grunting, hypoxemia, shallow breathing, irregular breathing/apnea
Gastrointestinal tract	Decreased feeding (refusal of bottle), poor latch after established breastfeeding, excessively long feeding times, fussiness	Vomiting, abdominal distension paralytic ileus and ulcerative necrotizing enterocolitis hypoglycemia or hyperglycemia, and metabolic acidosis.
Neurological	Incessant crying, hypotonia, thermal disturbances (hypothermia or fever), blunt neonatal archaic reflexes	Seizures, apnea, lethargy, encephalopathy, bulging fontanelle, features of meningitis
Renal	None	Oliguria
Hepatic/hematological	Hepatomegaly and direct hyperbilirubinemia in the absence of other known risk factors	Petechiae or bleeding suggestive of coagulopathy
Dermatological	New-onset rash, vesicles, erythema, or swelling around joints	Mottling and oozing umbilicus

To be clinically diagnosed, neonates should show symptoms in three different systems or the affection of two different systems showing clinical signs in the presence of a maternal risk factor [36]. Colorimetric analysis of skin color can also be used to assess the severity of sepsis [37]. Neonatal sepsis presents relatively vague findings as seen during the clinical assessment; thus, neonates are at risk of delayed recognition of sepsis until more serious clinical findings or vital sign abnormalities develop. This has led researchers to develop various “sepsis scores” by combining different clinical criteria and laboratory cutoff values of various markers and other variables. Despite the remarkable efforts, no unanimous scoring system has been selected to accurately define neonatal sepsis [38]. Early diagnosis is vital as neonatal sepsis can lead to an increased risk of neurocognitive sequelae including cognitive defects, visual/hearing impairments, or even detrimental outcomes such as cerebral palsy in patients treated with antibiotics [39]. As well as the development of atopic diseases in childhood has been linked to the earlier history of neonatal sepsis in that child [40].

Diagnosis

Concerning the subtle clinical findings, most neonates are at stake for delayed diagnosis of neonatal sepsis [41]. Up-to-date investigational modalities help aid in the early notification of neonatal sepsis and thus improve clinical outcomes [2]. Current methods of diagnosis include microbiological cultures, molecular techniques, hematological indices, and inflammatory biomarkers. Diagnostic tools and measured markers are summarized in Table 2.

**Table 2:** Common diagnostic indices and markers PCR: polymerase chain reaction, IL: interleukin, TNF: tumor necrosis factor, CD: cluster of differentiation.

Indices	Markers
Blood count	Total leukocyte counts, absolute neutrophil count, neutrophil ratio immature-to-mature neutrophil ratio, immature-to-total neutrophil ratio, platelet count
Acute phase reactants	C-reactive protein, procalcitonin, serum amyloid A, lipopolysaccharide-binding protein
Molecular diagnostics	Broad-range conventional PCR, real-time PCR, multiplex PCR, species- and genus-specific PCR, PCR followed by post-PCR processing, hybridization or mass spectrometry
Cytokines and chemokines	IL-6, IL-8, IL-16, TNF-alpha, soluble CD14, CD64, mannose-binding lectin, hepcidin

Microbiological Culture

Standard culture remains the “gold standard” that forms the basis of the diagnosis of neonatal sepsis [2]. Swabs from various sites including the umbilicus, pharynx, and rectum denote the causative organism and can also be used for antibiotic sensitivity [41]. The single most important factor that affects the identification of a pathogen is the volume of blood samples collected for culture [42]. Other factors include the time of blood collection [2] and maternal intrapartum antimicrobial exposure [43,44]. However, up to a third of newborns with meningitis may still show a negative blood culture [45]. The role of anaerobic culture is unclear; some studies state that anaerobic infections would be missed if strictly aerobic culture were used [46].

Molecular Techniques

Rapid testing diagnostic systems have greatly reduced the turnaround time for diagnosis of the organism [47] as they are less labor intensive, early targeted microbial therapy, have improved clinical outcomes, and lead to shorter hospital stays. Various types of molecular approaches have been developed over time, all of which primarily rely on the concept of amplification of bacterial 16S or 23S rRNA genes as well as the 18S rRNA gene of fungi [2,47].

The QuickFISH system takes a short reporting time frame period of about 20 minutes which is almost the same time used for Gram staining [2]. Other polymerase chain reaction (PCR)-based techniques include GeneXpert (one hour), FilmArray (one hour), and Verigene (2.5 hours) [2]. In contrast, the T2 magnetic resonance (MR) platform is an automated nanoparticle-based PCR assay that can detect *Candida* spp. in the blood with as few as 1 colony-forming unit (CFU)/mL within approximately three hours [48]. Moreover, peptide nucleic acid fluorescent in situ hybridization (PNA-FISH) molecular stain is a well-established methodology that uses a multicolor probe to differentiate *Enterococcus faecalis* from other enterococcal species within a three-hour time [49]. SeptiFast, SepsiTest, and PCR-amplified pathogen DNA are new diagnostic systems reported to have high diagnostic accuracy. SepsiTest is a commercial assay that can detect more than 300 pathogens but with a moderately slower change time (8-12 hours) [50].

Hematological Indices

Peripheral blood smear (toxic granulation, vacuolization, and Dohle bodies) and leukocytic count are conventionally used in diagnosing neonatal sepsis [2]. Absolute neutrophilic count <1,000/mm^3^ at ≥4 hours, i.e., neutropenia, is regarded as a precise indicator for early-onset neonatal sepsis [2]. Although the WBC count has limitations since its values are dynamic during the first 12 hours of life, serial measurements over 24 hours might be more informative than a single assessment [51]. A study by Sharma et al. found that leucopenia (WBC count <5,000/mm^3^) has low sensitivity (29%) but high specificity (91%) for diagnosing neonatal sepsis [22]. Other studies have shown that leukopenia is a better predictor than leukocytosis (WBCs > 20,000/mm^3^) after more than four hours [52]. The granulocyte monocyte colony-stimulating factor showed a high negative predictive value in one study when a cutoff level of 200 pg/mL was used [53]. While the most sensitive indicator is the immature-to-total neutrophil (I:T) ratio [22,54,55], variation still exists depending on gestational age and postnatal age [2]. Full-term neonates with an I:T ratio > 0.27 and preterm neonates with a ratio > 0.22 favor neonatal sepsis diagnosis [2].

Platelets can also be used to indicate sepsis. Sepsis causes a decrease in platelet production, and produced platelets are young and bigger in size resulting in an increase in the mean platelet volume (MPV). MPV indicates an increase in the diameter of produced platelets; therefore, an increase in MPV clinically indicates platelet production rate and activation [56]. Wang et al. concluded that there is an overall increase in the MPV in neonates with sepsis compared to healthy individuals [56]. Platelet distribution width (PDW) is also increased, indicating variability in platelet size. PDW increases in cases of platelet anisocytosis. There is a physiological correlation between both PDW and MPV, with both usually changing in the same direction. Platelet indices are useful in diagnosis and follow-up and can be used to assess the response to treatment [57].

Inflammatory Biomarkers

Serum C-reactive protein (CRP) elevation is directly correlated with the severity of illness. It rises within 10-12 hours and peaks after 36-48 hours in response to bacterial infections [2]. CRP and complete blood count (CBC) have a better negative predictive value that plays an integral role in early diagnosis [36]. At the early stages of infection, CRP has unsatisfactory low sensitivity [58,59]; therefore, periodic measurements of CRP at 24-48 hours alleviate its sensitivity and negative predictive value and are thus beneficial for supervising treatment [2]. It may be useful for surveilling treatment responses in neonates receiving antibiotic treatment. This implies that CRP may be more practically applicable for ruling out infection.

Another biomarker used is procalcitonin (PCT), it is a prohormone of calcitonin without hormonal activity, and it is synthesized from the *CALC-I* gene on chromosome 11 during periods of inflammation [60]. PCT is inhibited by interferon, a cytokine frequently generated in viral infections [60-62]. Hence PCT has surfaced as a viable biomarker to distinguish between viral and bacterial etiologies. Despite PCT being of greater sensitivity (five- to 20-fold increase from baseline) compared to CRP (three- to eight-fold increase from baseline) in sepsis, it is less specific due to its physiological increase in newborns during the first few days of life [33]. Therefore, a periodic PCT measurement at 24 hours of age may be more useful for making an early diagnosis and reducing the length of antibiotic therapy [63]. The sensitivity and specificity of PCT were shown to be 81% (95% CI: 74-87%) and 79% (95% CI: 69-87%), respectively, in a meta-analysis of 1,959 patients [64].

Serum amyloid A (SAA), a chemoattractant, levels increase 1,000 times higher than normal basal levels in the presence of an infection or injury but are also largely dependent on the patient’s liver function and dietary and health status [65]. SAA levels in infants with sepsis were notably high (p < 0.01) when contrasted with healthy infants at 0-8-24 hours [66]. SAA has an overall better diagnostic value in predicting early-onset neonatal sepsis at all time points relative to CRP [2]. High levels of pro-adrenomedullin are also associated with increased neonatal mortality due to sepsis [67].

Some interleukins such as IL-6 and IL-8 are known to be “early warning biomarkers” in a few countries [68-71]. Early cases of neonatal sepsis were shown to have elevated IL-6 levels measured from the umbilical cord [72]. IL-6 levels rise early immediately after bacteremia before the rise in CRP levels, as an early biomarker having a higher sensitivity. In preterm neonates with EOS, an increase in umbilical plasma levels of cytokines (tumor necrosis factor (TNF)-alpha, CRP, IL-1b, IL-6, IL-8, p55, p75, and IL-1 receptor antagonist) has been noted to occur in prenatal immune response [2]. IL-8 increases within one to three hours with a reported specificity and sensitivity of 84% and 78%, being similar to CRP [2]. Nevertheless, cytokine lab measurement is time-consuming and nonpractical due to the cost of the immunoassays [73]. The combination of IL-6, PCT, and soluble triggering receptor expressed on myeloid cell-1 (sTREM-1) is favorable because every biomarker forms a different constituent in the pathophysiology of sepsis [2].

Newer Diagnostic Technique

Newer diagnostic techniques include microarray technology which is characterized by the hybridization of samples on either a glass or silicon slide that was foreloaded with either an array of protein or nucleic acid products. This preparation allows us to concurrently detect pathogens and microbial virulence and permits us to unravel the host immune response profile. Although it is highly sensitive and specific, it requires specialized instruments and highly trained staff to operate on [74,75]. Another technique currently being used is matrix-assisted laser desorption-ionization/time-of-flight (MALDI-TOF) mass spectroscopy which can report results within 30 minutes [2]. Quantitative real-time PCR (qPCR) amplification systems are also being used; they have a high negative predictive value and quick results [41].

Other new methodologies include lipopolysaccharide-binding protein [76], volatile organic compounds [77], soluble triggering receptor expressed on myeloid cell-1 [78], presepsin [79], CD64 [80], CD11b [81], and S100 protein A12 [82]. Such diagnostic tools are promising and prove to be more rapid and sensitive indicators of the disease [2].

Treatment

Treatment for neonatal sepsis aims to provide supportive care for any potential organ dysfunction, along with the administration of IV fluids, antibiotics, antiviral agents, and antifungal medications [83]. Depending on the condition severity, additional interventions may be required such as oxygen supplementation and admission to the NICU, and the addition of vasoactive amines may be mandatory. EOS and LOS have very distinguishable causes and are caused by different microorganisms, as a result, they have different treatment strategies [41]. Treatment choice, empirical or definitive, is proposed depending on several factors type of sepsis either EOS or LOS, if the cause is nosocomial infection or a community-acquired infection, or if there are any comorbidities present. Other factors that may alter treatment are if the cause is prolonged rupture of membranes, if amniotic fluids have a foul odor, and vaginal colonization, among others. These factors play a role in helping identify the best treatment option for the patient and determining the most suitable fitting antimicrobial therapy [20,41].

Treatment duration varies from one patient to another, with some patients requiring a prolonged duration of three weeks if cultures indicate positive results for blood or cerebrospinal fluid (CSF). However, some researchers suggest that a 10-day treatment duration is adequate in patients with pathogen-clear laboratory tests [18,19,41,84]. Till date, there is no unison on the optimal treatment duration for antibiotic regimens in neonatal sepsis.

Treatment of early-onset neonatal sepsis

There are several microorganisms that have been commonly implicated in EOS including Group B Streptococcus (GBS), *Escherichia coli*, *Haemophilus influenzae*, and *Listeria monocytogenes*. Vertical transmission has a very significant role in the route of infection involved in EOS [85,86]. When sepsis is suspected, it is important to obtain culture samples and initiate empirical antibiotic treatment. The preferred empirical treatment for EOS consists of a combination of aminoglycoside (typically gentamicin) and ampicillin. Renal function should be monitored in newborns receiving these medications. If gram-negative meningitis is suspected, a third- or fourth-generation cephalosporin should be added to the regimen [85]. Although cephalosporins are not effective against *Listeria monocytogenes*, a combination of a third- or fourth-generation cephalosporin and ampicillin can be considered. In cases where newborns have been previously treated with cephalosporins, a carbapenem antibiotic may be an alternative, taking into account local resistance patterns [84]. In neonatal intensive care units, the combination of piperacillin, tazobactam, ampicillin, and sulbactam is increasingly being used. Ampicillin in combination with sulbactam and piperacillin combined with tazobactam are used to treat intraamniotic infections [87]. If meningitis is suspected, it is recommended to add a beta-lactamase inhibitor along with ampicillin [85].

Vancomycin is recommended when Staphylococcus infection is suspected, particularly in cases associated with cephalosporin use (third or fourth generation). In situations where a fungal infection is suspected, aggressive empirical treatment with amphotericin B is recommended [41]. The most common fungus associated with neonatal sepsis is Candida, but it is crucial to consider other pathogens such as aspergillosis, cutaneous and intestinal zygomycosis, trichosporonosis, cryptococcosis, and others [88]. Upon pathogen identification, treatment is guided by an antibiogram (Table 3).

**Table 3:** Common causative organisms in early-onset sepsis (EOS) and treatment

Antibiotic (Considering Antibiogram)	Pathogen
Aminoglycosides (gentamicin) + ampicillin	Group B streptococci, *Listeria monocytogenes*, gram-negative enteric bacteria
Gentamicin + penicillin	Group B streptococci
Penicillin (aminoglycoside-synergism)	Enterococci
Vancomycin	Ampicillin-resistant Enterococcus
Vancomycin	Methicillin-resistant *Staphylococcus aureus*, coagulase-negative Staphylococcus
Cephazolin	Methicillin-sensitive *Staphylococcus aureus *
Cephalosporin (third/fourth generation)	Methicillin-sensitive *Staphylococcus aureus* involving central nervous system
Cefepime	Pseudomonas species
Carbapenem, cefepime	Enterobacteriaceae extended-spectrum beta-lactamases
Clindamycin, ampicillin + tazobactam or metronidazole	Anaerobic infections
Metronidazole	Anaerobic infection involving central nervous system
Amphotericin B deoxycholate	Fungal infection
Fluconazole (alternative treatment, guided by antibiogram)	Fungal infection

Intravenous immunoglobulins (IVIGs) can be beneficial in the treatment of neonatal sepsis. A retrospective study conducted showed that administering IVIG, mainly IgM-enriched immunoglobulins, exhibited higher antimicrobial activity compared to IgG. Immunoglobulins improve immune function and therefore are beneficial [89]. Another randomized clinical trial concluded that the use of granulocyte colony-stimulating factor in preterms resulted in decreased mortality. It was especially effective in patients with neonatal sepsis and neutropenia [90].

Treatment of late-onset neonatal sepsis

LOS is frequently associated with gram-positive microorganisms, particularly coagulase-negative *Staphylococcus aureus* (76%). Gram-negative microorganisms, fungal species like Candida, and viruses such as rhinovirus and respiratory syncytial virus (RSV) are also commonly implicated in LOS [18]. Guidelines recommend the use of a beta-lactam, specifically benzylpenicillin or ampicillin, combined with aminoglycosides, mainly gentamicin [18]. Typical empiric antibiotic regimens for neonatal sepsis often include ampicillin, a third-generation cephalosporin, along with an aminoglycoside or vancomycin. Most guidelines recommend the use of a beta-antibiotic with an aminoglycoside; this would usually include ampicillin, flucloxacillin, or penicillin combined with gentamicin [18].

Alternatively, a combination of flucloxacillin and gentamicin can be effective in treating most cases caused by other organisms [41]. In situations involving necrotizing enterocolitis, clindamycin or metronidazole may be added to cover anaerobic bacteria. Cefotaxime is commonly reserved for infants with meningitis, while it is advisable to avoid ceftriaxone due to potential complications such as hyperbilirubinemia and the formation of calcium-ceftriaxone crystals [83]. The choice of antibiotic therapy should be based on culture results to ensure targeted treatment.

Newborns who have risk factors for Candida sepsis should be administered empiric antifungal therapy [41]. To prevent invasive fungal infections, sepsis care bundles, which include measures such as reducing the duration of central catheter use, practicing proper hand hygiene, implementing antibiotic stewardship programs, and considering prophylactic use of fluconazole in high-risk infants, are recommended [41]. In cases of sepsis caused by Enterobacter, Serratia, or Pseudomonas, treatment with a combination of a beta-lactam or beta-lactamase inhibitor and an aminoglycoside is advised [20].

For preterms with systemic extended-spectrum beta-lactamase infections, meropenem is recommended as a treatment option. A study has shown that a prolonged intravenous infusion of meropenem, administered over a period of four hours every eight hours, resulted in improved clinical outcomes for neonates with gram-negative LOS compared to the standard strategy of administration over 30 minutes every eight hours [41]. However, it is important to note that the overuse of antibiotics can have negative effects on the microbiome, contribute to organ dysfunction, and may lead to idiosyncratic toxicities. It is vital to adhere to infection control protocols as they are the foundation of preventing LOS.

Preventing neonatal sepsis

In order to prevent neonatal sepsis, several strategies have been put into place. First, maintaining adequate hand hygiene is necessary, this can be achieved by frequently washing hands and using antiseptic alcohol rubs in order to decrease infectious agent transmission. Another important measure is the early initiation of trophic enteral feeding, which stimulates the gastrointestinal tract, supports intestinal maturity, and prevents bacterial translocation [86]. Breastfeeding is highly encouraged as it provides essential components like immunoglobulin A (IgA), fatty acids, and amino acids that enhance the infant's immune system. The use of probiotics, particularly Bifidobacterium and Lactobacillus species, is still being studied for their potential to improve the gut microbiome and immune function. Additionally, lactoferrin, a protein found in human milk, shows promise in reducing the incidence of neonatal LOS, especially when combined with probiotics. However, further research is needed to establish the effectiveness and safety of lactoferrin. Therefore, the routine prophylactic use of lactoferrin cannot be recommended until more evidence is available [85].

## Conclusions

Newborn sepsis mortality rate varies enormously depending on the hospital and across countries. Worldwide it is estimated that 1.3 million cases of neonatal sepsis happen annually and the consequence of 203,000 deaths per year births. Changing this reality is a difficult challenge, but one must be undertaken. Preventive measures, early diagnosis, and establishment of efficient management are the only ways to avoid septic shock, multiple organ failure, and consequently death. Early diagnosis of neonatal sepsis provides physicians with time to determine the cause/causative organism which helps to provide more efficient treatment in a more adequate time preventing the occurrence of further complications.
